# IMPLEMENTING A STAKEHOLDER AND POLICY MAP TO TRACK FEDERAL CAREGIVER POLICY ACTIVITY

**DOI:** 10.1093/geroni/igad104.1643

**Published:** 2023-12-21

**Authors:** Michael Wittke

**Affiliations:** National Alliance for Caregiving, Washington, District of Columbia, United States

## Abstract

A top priority for family caregiver policy should be a focus on accountability and transparency regarding federal actions identified within the National Strategy to Support Family Caregivers. It is crucial to have accountability and transparency as the National Strategy is being updated and refined over the course of time that occurs between the release of the initial strategy and the publishing of the next version of the strategy. Increased accountability and transparency are especially important as stakeholders continue to champion and build on the foundation that has been established in the process of creating the 2022 National Strategy to Support Family Caregivers. Establishing outcomes measures will also inform how the stakeholder community may shift their priorities and operations to align with successes and challenges outlined in an updated National Strategy. To ensure that accountability and transparency are at the forefront of public interest, and as agency leadership communicates the intended outcomes of their critical contributions to America’s family caregivers, it is essential to establish a process to communicate the meaningful impact regarding federal actions on this population. As the National Strategy is being examined for future updates, NAC has facilitated a systematic process to map the actions that federal agencies have identified as core priorities and track their progress toward implementation. That mapping process will be revealed and analyzed during this presentation.

